# Cost-Minimization Analysis Favours Intravenous Ferric Carboxymaltose over Ferric Sucrose for the Ambulatory Treatment of Severe Iron Deficiency

**DOI:** 10.1371/journal.pone.0045604

**Published:** 2012-09-21

**Authors:** Xavier Calvet, Miquel Àngel Ruíz, Angelina Dosal, Laura Moreno, Maria López, Ariadna Figuerola, David Suarez, Mireia Miquel, Albert Villoria, Emili Gené

**Affiliations:** 1 Servei Aparell Digestiu, Hospital de Sabadell, Institut Universitari Parc Taulí, Universitat Autònoma de Barcelona, Sabadell, Spain; 2 CIBERehd, Instituto de Salud Carlos III, Barcelona, Spain; 3 Departament de Comptabilitat Analítica, Hospital de Sabadell, Sabadell, Spain; 4 Unitat d’Epidemiologia i Avaluació-Fundació Parc Taulí, Sabadell, Spain; 5 Servei d’Urgències Hospital de Sabadell, Institut Universitari Parc Taulí, Universitat Autònoma de Barcelona, Sabadell, Spain; Groningen Research Institute of Pharmacy, United States of America

## Abstract

**Objective:**

Intravenous iron is widely used to treat iron deficiency in day-care units. Ferric carboxymaltose (FCM) allows administration of larger iron doses than iron sucrose (IS) in each infusion (1000 mg vs. 200 mg). As FCM reduces the number of infusions required but is more expensive, we performed a cost-minimization analysis to compare the cost impact of the two drugs.

**Materials and Methods:**

The number of infusions and the iron dose of 111 consecutive patients who received intravenous iron at a gastrointestinal diseases day-care unit from 8/2007 to 7/2008 were retrospectively obtained. Costs of intravenous iron drugs were obtained from the Spanish regulatory agencies. The accounting department of the Hospital determined hospital direct and indirect costs for outpatient iron infusion. Non-hospital direct costs were calculated on the basis of patient interviews. In the pharmacoeconomic model, base case mean costs per patient were calculated for administering 1000 mg of iron per infusion using FCM or 200 mg using IS. Sensitivity analysis and Monte Carlo simulation were performed.

**Results:**

Under baseline assumptions, the estimated cost of iron infusion per patient and year was €304 for IS and €274 for FCM, a difference of €30 in favour of FCM. Adding non-hospital direct costs to the model increased the difference to €67 (€354 for IS vs. €287 for FCM). A Monte Carlo simulation taking into account non-hospital direct costs favoured the use of FCM in 97% of simulations.

**Conclusion:**

In this pharmacoeconomic analysis, FCM infusion reduced the costs of iron infusion at a gastrointestinal day-care unit.

## Introduction

Patients with digestive conditions such as chronic liver disease or inflammatory bowel disease frequently suffer from chronic iron losses that require large doses of supplemental iron. In these patients, oral iron administration is often not feasible or sufficient because intestinal absorption and digestive tolerance of iron salts are poor [1]. By contrast, intravenous (i.v.) iron is usually well tolerated and allows administration of larger iron doses [2]. Many studies have shown that i.v. iron is more efficacious and better tolerated than oral iron supplementation [1]; [3]–[8]. In addition, in patients with inflammatory bowel disease it remains unclear whether large oral iron doses may induce disease flares or may even increase cancer risk [9].

In the past, iron dextran formulations carried a significant risk of anaphylaxis. For this reason, new formulations such as iron sucrose (IS) and, later, ferric carboxymaltose (FCM) were developed [10]. These compounds have shown an excellent safety profile and IS was the preferred drug in most hospitals until FCM became available. Although both compounds allow administration of much larger daily doses than their oral counterparts, FCM allows the administration of up to 1000 mg of iron in a single infusion, while IS administration is restricted to a maximum of 200 mg per day [11]. Although FCM is more expensive than other i.v. iron preparations, the ability to administer higher doses has a clear advantage for both patients and day care units, as fewer hospital visits and vein punctures are required [12]–[13]. It is important, however, to determine the comparative total costs of both strategies from the hospital and societal perspectives in order to determine which infusion strategy is preferable.

**Figure 1:**
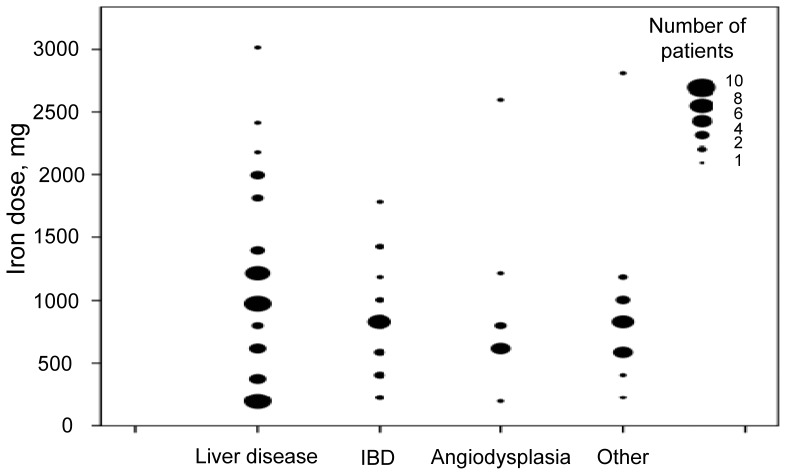
Iron dose according to the intravenous iron indication. The size of the dots corresponds to the number of patients receiving a given dose of intravenous iron. The most frequent indication for intravenous iron was chronic blood losses associated to liver disease. These patients also required the highest doses of intravenous iron.

The aim of this study was to compare the cost implications of using i.v. FCM versus IS – at present the most used i.v. therapy – for treating iron deficiency in a specialized day-care unit devoted to digestive diseases, using a cost-minimization analysis.

**Table 1:** Indication, mean dose, transfusion requirements and mortality according to the indication of intravenous iron.

Indication	PatientsN (%)	Iron dose (mg)mean ± SD	Patients transfusedn (%)	Blood Unitsmean ± SD	Mortalityn (%)
**Chronic liver disease**	55 (49.5)	1178±138	29 (53)	6.1±0.9	18 (32.7)
**Inflammatory bowel disease**	22 (19.8)	800±84	3 (14)	3.3±0.3	2 (9)
**Angiodysplasia**	12 (10.8)	833±174	8 (67)	3.5±1.2	2 (16.7)
**Other**	22 (19.8)	864±106	6 (27)	4±1.1	1 (4.5)

## Patients and Methods

The records of 111 consecutive patients receiving i.v. iron at the gastrointestinal diseases day-care unit of the Hospital de Sabadell, Barcelona, Spain, from August 2007 to July 2008 were retrospectively reviewed. Data on the number of infusions and the amount of iron administered per patient were collected. The full description of the series of patients has been published elsewhere [14]. Costs of drugs were obtained from the prices approved by the Spanish Agency for the Regulation of Drugs and Healthcare Products [15]. All personnel and indirect costs were obtained from the accounting department of the Hospital of Sabadell. The Hospital’s accounting department determines the direct and indirect costs for outpatient iron infusions by using a full-cost model for assigning costs to each process. This model attributes all corresponding organizational costs to any process or product whose cost one intends to measure. The cost of the product or the process includes direct and indirect costs. Direct costs include the needs for medical material, personnel and diagnostic procedures. In this case cost of personnel was calculated including all the staff working part or full time in the day-care unit, and included nursing and auxiliary personnel plus part-time medical surveillance. Indirect costs include the fraction of the common hospital costs imputable to a process and include (among other things) administrative costs, structural costs, and maintenance and cleaning services. This is performed by dividing the hospital into different processes and sub-processes with their corresponding direct and indirect cost assignation. In addition to direct and indirect costs, non-hospital direct costs were calculated by asking a consecutive unselected series of 605 patients –297 female, mean age 50±25 years, of whom 161 were actively working – about the costs associated with travelling to the hospital and missed working hours. Costs were measured in € for the year 2009. Costs were not discounted due to the short time frame of analysis.

**Table 2:** Costs, including upper and lower bounds used in the sensitivity and Monte Carlo analysis.

Cost (2009 €)	Baseline	Lower bound	Upper bound
**Cost iron sucrose 200 mg**	23	5	23
**Cost Ferric carboxymaltose 1000 mg**	200	80	200
**Cost infusion iron sucrose (1** **h 15')**	47	35	60
**Cost infusion Ferric carboxymaltose (45')**	35	28	42
**Indirect cost/h**	6	2	10
**Staff cost/hour in day-care unit**	18	12	24
**Cost of infusion devices**	7	5	10
**Non-medical direct costs/session**	10	2	20

Costs of drugs were obtained from the prices approved by the Spanish Agency for the Regulation of Drugs and Healthcare Products (15). All personnel and indirect costs were obtained from the accounting department of the Hospital of Sabadell.

A pharmacoeconomical evaluation by using cost minimization analysis was performed from hospital and societal perspectives, the latter including non-hospital direct costs. Cost-minimization is a tool used in pharmacoeconomics and is applied when comparing multiple drugs of equal efficacy and equal tolerability. Theoretical base case total costs per patient were calculated using the individual patients’ data for two scenarios: 1) administering the amount of iron required for each patient with 1000 mg of iron per infusion using FCM or 2) administering 200 mg of iron per infusion using IS. For the FCM infusion, the dose was rounded to the next 1000 mg dose. Thus, a patient who received a total dose of 800 mg of iron was assumed to receive four doses of IS or one 1000 mg infusion of FCM and a patient receiving 1400 mg of IS was assumed to receive two 1000 mg infusions of i.v. iron. Primary outcome measure was the cost of infusion per patient in one year. This parameter was calculated by adding: 1) the cost of intravenous iron, 2) direct hospital costs (personnel, infusion material) and 3) indirect hospital costs (the general functioning costs) and 4) direct non-hospital costs (travel, time off work for the patient and – if applicable – the accompanying person). Calculations were performed using Microsoft Excel XP™.

**Figure 2:**
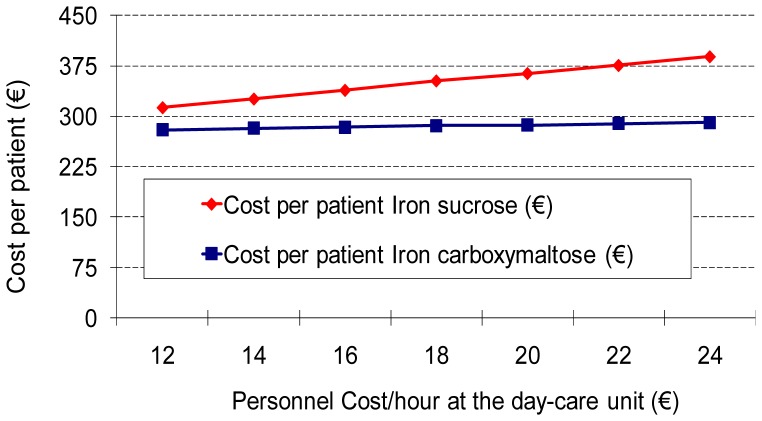
One-way sensitivity analysis: Influence of the staff cost/hour in the total cost per patient. Incremental cost of iron sucrose over Ferric carboxymaltose increases as the staff costs increase.

One-way and two-way sensitivity analyses were performed by changing baseline estimates for costs within a range of potentially reasonable values and evaluating whether these changes modify the conclusions reached using baseline estimates for costs. Finally, probabilistic sensitivity analysis was performed using Monte Carlo simulation for Microsoft Excel XP™. Monte Carlo simulations are a class of computational algorithms that rely on repeated random sampling to compute their results. The model assigns random values under a predetermined distribution to the variables shown to be more influential in the final results in the one and two-way analyses. Variables included in the analysis were cost of IS and FCM, indirect costs/hour, staff cost per hour, cost of infusion devices and non-medical direct costs. These variables were tested both according to a normal distribution and a uniform distribution, and performing 10,000 iterations.

**Figure 3:**
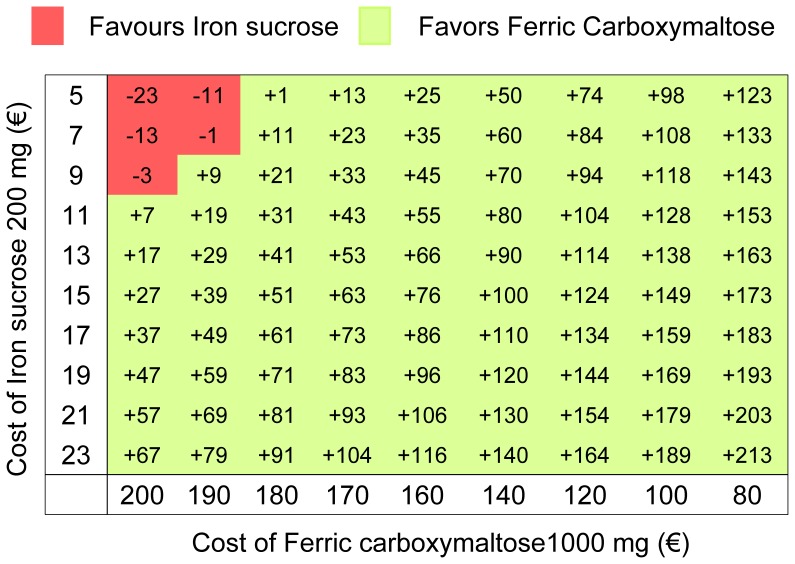
Two-way sensitivity analysis modifying costs of ferric carboxymaltose and iron sucrose. The values in the table show that the incremental cost (€) per treated patient was favoured ferric carboxymaltose, unless if it cost more than €180 per 1000 mg and if cost of iron sucrose decreased simultaneously below €10 per 200 mg.

The Ethics Committee of the Hospital of Sabadell reviewed and approved the study. The study used retrospective data and neither intervention nor genetic testing was performed. Patients’ data were processed anonymously and were not available to third parties. As a result, and in accordance with Spanish legislation, the Ethics Committee did not require informed consent for the study. The 2007 Biomedical Research Act established the need for informed consent and Ethics Committee approval for medical investigations that either performed genetic testing or required invasive procedures (Articles 1, 4.1 and 16 of the Law) but not for retrospective studies. In fact, at present, there is no specific legal regulation for the retrospective analysis of data obtained from clinical records. These studies must, however, comply with the 1999 Personal Data Protection Act.

**Figure 4:**
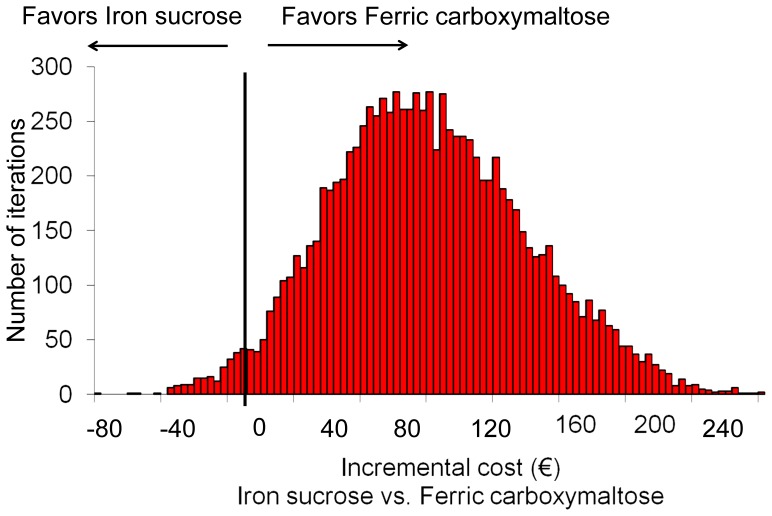
Monte Carlo simulation after 10,000 iterations. The bars show the number of iterations that resulted in a given incremental cost value. Values under 0 correspond to the simulations that favour the use of Iron sucrose. As shown by the figure, the model favoured the use of ferric carboxymaltose in 97% of the iterations.

## Results

A hundred and eleven patients (46 (41%) female, mean age 63.8±18) were included in the previous clinical study. These 111 patients received 557 IS infusions, with a mean dose of 1033±810 mg (range 200 to 5200 mg) of iron per patient. Major indications for iron infusion were iron-deficient anemia associated with liver cirrhosis, inflammatory bowel disease or gastrointestinal angiodysplasia. Table 1 shows the number of patients, mean iron dose, the number of patients requiring transfusion and the mean transfusional requirements for the major indications for transfusion. In general patients requiring intravenous iron had severe baseline disease. Thirty-four (31%) were severely ill (category IV in the American Society of Anesthesiologists – ASA – Physical Status classification). Another 30 (27%) were ASA III, 26 (23%) ASA II and only 21 (19%) did not have significant baseline disease (ASA I). Seventy-one of the patients (64%) required admission. The mean number of admissions during the study period was 4.2, a third of them directly related to anemia. Twenty-three patients (21%) died during the study period, all due to complications of their baseline disease and most due to complications of their chronic liver disease. Anemia was considered to contribute significantly to death in seven patients. The doses of iron according to the indication in the clinical retrospective data collection are shown in figure 1. Major cost estimates are shown in table 2.

As patients were assumed to receive iron infusion rounded to the next 1000 mg dose when FCM was given, the calculated total amount of iron given under base case assumptions was higher by using FCM (135 g) than the amount given by using IS (111 g). The estimated total cost of iron infusion per patient was €303.6 for IS and €273.9 for FCM. So, the incremental cost of using IS was €30 per patient. When the non-hospital direct costs were added to the model, the difference in favour of FCM increased (€353.8 for IS vs. €286.5 for FCM with an incremental cost of €67).

In one-way sensitivity analysis, IS and FCM prices and the day-care unit personnel cost per hour were the most influential variables in the model. Regarding personnel costs, the difference in favour of FCM increased as the personnel cost increased (Figure 2). However, even when decreasing the personnel costs by 50%, the analysis remained favourable to FCM. A two-way sensitivity analysis changing at the same time the costs of IS and FCM showed that, in the current scenario, the price of IS had to be reduced to less than 9 euros to make the use of this drug less expensive than the use of FCM (Figure 3). The analysis also showed that a small decrease in the cost of FCM would lead to cost savings independently of the IS cost. Similarly, the Monte Carlo simulation favoured the use of FCM in 97.2% of all possible scenarios, with a mean incremental cost of €87 for the use of IS (Figure 4). Results were similar regardless of the assumed distribution (uniform or normal) of the variables.

## Discussion

The present cost minimization analysis evaluates the comparative cost of using either IS or FCM for treating iron deficiency anemia in a Gastrointestinal Diseases day-care unit. The study suggests that under standard conditions the savings in costs associated to the infusion and the day-care visits offset the higher drug cost of FCM when compared with IS. The results of the analysis were fairly stable and were reproduced in most of the univariate and multivariate sensitivity analyses performed. It is worth noting that these conclusions were obtained using the Spanish National Health System personnel costs, which are particularly low compared to those of other Western European countries. The savings due to the reduced number of infusions can be expected to be greater in situations with higher personnel costs.

Our results are mostly in accordance with the only similar study published [16]. In that study, Bager et al. used three different pharmacoeconomic approaches to compare the use of FCM and IS for iron repletion, in patients with inflammatory bowel disease. Both a cost-effectiveness analysis and a cost-benefit analysis favoured FCM. However a budget impact analysis from a hospital perspective favoured IS. By contrast, cost-minimization analysis in our study, which is roughly equivalent to the budget impact analysis in Bager’s manuscript, favoured FCM. There are two main reasons for the difference. Firstly, drug costs in Denmark are twice as high as those in Spain, decreasing the potential advantage of FCM. Secondly, and probably more importantly, Bager’s study presumably underestimated personnel costs as only nursing time was taken into account. Although mean salaries in Spain are less than half of those in Denmark [17], our estimated personnel costs were higher than those reported by Bager et al. probably because we included the cost of auxiliary personnel and the medical staff support. Taking these two factors together, our analysis was largely favourable to FCM. In addition, when non-hospital direct costs were included, FCM was favoured even more.

The analysis is based on iron doses administered to real patients. This is both a strength and a limitation of the study. It represents a strength because the estimated costs will probably reflect the actual situation in clinical practice. It can be seen as a limitation because the analysis was performed in a particular patient population, most of them of advanced age and suffering from a range of digestive diseases leading to iron deficiency [14]. The costs could be different in another setting such as, for example, monographic inflammatory bowel disease units where patients are younger and most of them are of working age, which increases work time losses and therefore non-medical direct costs. In any case, the larger the non-hospital direct costs, the more FCM is favoured.

A second limitation is that the analysis assumes equal efficacy and safety for both drugs. Regarding efficacy, we reasonably assumed that the efficacy of a given dose of iron would be similar regardless of whether the dose was single or multiple. In fact, very recent data from controled trials comparing IS and FCM in patients with IBD suggest that FCM may be more efficacious than IS. If confirmed, this superior efficacy could give additional arguments suporting the clinical use of FCM [13].

It also seemed reasonable to analyze the strategy of administering repeated 1000 mg iron doses to patients with chronic and continuous iron losses that would require periodical iron infusions. However, in order to model this approach we had to round the iron dose to the next 1000 mg multiple. For this reason, the iron dose administered in the FCM arm in the model was superior to those administered in the IS model, increasing the drug costs in the FCM groups. As the efficacy in both arms was assumed to be similar, this may have introduced a slight bias in favour of the 200 mg dose IS infusion.

Regarding safety, early safety reviews showed a trend towards increased mortality in patients receiving FCM, which led to a request for further data from the FDA [18]. However, the trend towards increased mortality did not correlate with an increase in any other type of complication and was due to a wide range of causes unlikely to be related to iron treatment [18]. In addition, further studies and reviews have not found any risk increase to be associated with FCM [19]–[22]. The most frequent cause of death (5 of 11 deaths in randomized trials) was cardiac disease. A recent study in patients with cardiac failure found an improvement in quality of life and functional status along with a trend towards lower complications and mortality in patients treated with FCM [23], thus essentially ruling out an increased risk of death or complications attributable to FCM administration. Finally, our study confirms that administering larger doses of iron in fewer infusions reduces both hospital and societal costs. However, its scope is limited to direct and indirect costs. Reducing outpatient visits and infusions is expected to improve quality of life and has the potential to reduce hospital admissions due to better ambulatory control [23], [24]. Measuring and adding these benefits to the cost advantage shown in the present study could further increase the estimated benefits of FCM [16].

Iron deficiency secondary to chronic diseases is increasing. Indications for i.v. iron use are expanding as there is growing evidence of reduced requirements for blood transfusions and improved quality of life [23], [24]. Intravenous iron administration has already proven to be clinically effective in correcting iron deficiency in a wide range of clinical settings, from inflammatory bowel disease to heart failure [1], [11], [18], [23]–[25]. Data on the efficacy of intravenous iron in other settings are, however, scarce. Previous studies by our group have shown an extensive use of i.v. iron for indications such as chronic blood loss associated with liver disease or angiodysplasia [14]. The efficacy of intravenous iron (and specifically of, FCM in these indications remains to be determined.

In conclusion, in this pharmacoeconomical model, ferric carboxymaltose infusion was less costly than iron sucrose infusion and appeared to reduce the costs of i.v. iron treatment in outpatient day-care units.
